# Are Risk Factors Common to Thyroid Cancer and Nodule? A Forty Years Observational Time-Trend Study

**DOI:** 10.1371/journal.pone.0047758

**Published:** 2012-10-31

**Authors:** Angelo Carpi, Giuseppe Rossi, Rossana Romani, Giancarlo Di Coscio, Andrea Nicolini, Tommaso Simoncini, Matteo Russo, Jeffrey Mechanick

**Affiliations:** 1 Department of Clinical and Experimental Medicine, University of Pisa, Pisa, Italy; 2 CNR, Institute of Clinical Physiology, Unit of Epidemiology and Biostatistics, Pisa, Italy; 3 Department of Oncology-Pathology, University Hospital, Pisa, Italy; 4 Department of Experimental Medicine, Sapienza University of Rome, Rome, Italy; 5 Department of Cellular and Molecular Pathology, IRCCS San Raffaele Pisana, Rome, Italy; 6 Division of Endocrinology, Diabetes and Bone, Mount Sinai School of Medicine, New York, New York, United States of America; The Chinese University of Hong Kong, Hong Kong

## Abstract

A progressive increase in the incidence of thyroid cancer (TC) has been reported over the last few decades. This either reflects the increased number of newly discovered and accurately selected thyroid nodules with more sensitive technologies and a relative more potent carcinogenic effect of pathogenetic factors in malignant, but not benign nodules. This observational time-trend study addresses this issue by analysing the proportion of TC within 8411 consecutive thyroid nodule (TN) patients evaluated in Pisa by the same pathology Department and individual clinician over a four-decade period. From 1972 to 1979 surgery was used to detect TC among the TN patients: 1140 TN patients were operated on and 35 cancers were detected (3.1% of all the TN patients). Subsequently, needle aspiration techniques were used to select TN for surgery. From 1980 to 1992, 5403 TN patients were examined, 483 were selected for surgery, and 150 cancers were found (2.8% of all the TN patients). From 1993 to 2010, 1568 TN patients were examined, 143 were selected for surgery, and 46 cancers were found (2.9% of all the TN patients). Therefore, in the University Hospital of Pisa, and independent of preoperative TN selection protocols, these proportions of TN eventually found to harbor TC remained statistically unchanged over 40 years (p = 0.810). This finding suggests that pathogenic risk factors and more sensitive diagnostic technologies did not differentially affect the incidence of TN and TC.

## Introduction

A progressive increase in the incidence of thyroid cancer (TC) has been reported in various countries over the last few decades [1]–[3].

The hypotheses to explain these epidemiological data include the increasing use of procedures with progressively increasing sensitivity for diagnosing TC, as well as an increased exposure to environmental factors, such as ionizing radiation [1]–[4]. In the former case, compared with palpation alone on physical examination, the increased detection of TC within a thyroid nodule (TN) has been facilitated with the use of needle aspiration techniques (NAT; including fine-needle aspiration [FNA] for cytological examination; large-needle aspiration biopsy [LNAB] for histological examination and, if available, molecular analyses; and ultrasound [US]-guidance) [1]–[7].

Therefore, the question arises whether the increased TC incidence is due to the improved NAT or rather, some increased or more potent effect of carcinogenic factors specific for malignant, but not benign TN [1]–[3]. This retrospective study addresses this issue by analyzing the proportion of TN harboring a TC during a four-decade period at the University Hospital of Pisa.

## Methods

### Thyroid nodule patients and cancer detection modality

This observational time-trend study includes a total of 8411 consecutive patients with palpable TN that were all evaluated by the same clinician (AC) and Pathology Department from 1972 to 2010 at the University Hospital of Pisa. The patients have been divided into 3 groups based on 3 consecutive investigative periods. The first group [5] consists of patients with palpable TN evaluated in the Nuclear Medicine Department from 1972 to 1979 when clinical history, physical examination, and iodine-131 thyroid scans were the principal methods used to advise euthyroid patients with a palpable TN to have surgery or use thyroid hormone suppression therapy. Surgical excision was advised when the TN did not decrease in size after some months of therapy. Thus, the vast majority of euthyroid patients with palpable TN were treated surgically. 1,140 patients, 185 men (16.2%) and 955 women (83.8%), with ages 14 to 79 years, underwent surgery. A single TN was palpable in 815 patients (71.5%) and multiple nodules were palpable in 325 (28.5%).

The second group previously reported [5] consists of patients with palpable TN examined from 1980 to 1992. In these patients, NAT were mainly applied to detect cancer in order to avoid needless surgery on the majority of patients who have benign nodules. Both the low prevalence of malignancy in patients with nodular thyroid disease and the relatively benign nature of thyroid cancer led to the referral of only higher- risk patients for thyroidectomy [5]. This group consisted of a total of 5.403 patients, 651 men (12%) and 4.752 women (88%), with ages 14 to 80 years. A single TN was palpated in 3,943 subjects (73%), while 1,500 had multiple TN (27%). All of these patients underwent FNA with cytologic examination; 1.668 (31%) were also examined by LNAB to provide additional preoperative histologic information [5]. 483 (8.9%) patients were then selected for surgery.

The third unpublished group consisted of 1,568 patients with a TN examined from 1993 to 2010, a period of time where other commitments limited the clinical case load of the clinician (AC). There were 213 men (14%) and 1,355 women (86%), aged 12 to 87 years. A single nodule was palpated in 1,053 subjects (67%), while 515 had multiple nodules (33%).

All of these patients underwent FNA with cytology examination; 514 (32.7%) were also examined by LNAB histology. 143 (9.2%) patients were selected for surgery.

All of the non-operated patients in the three groups underwent long-term clinical follow-up.

The study was approved by Committee of the Department of Reproduction and Ageing, Pisa University. Verbal consent was obtained because LNAB is part of our routine work since 1980. A survey documented the process. Verbal informed consent was obtained from all participants. Verbal consent procedure was approved by Ethics Committee of the Department of Reproduction and Ageing, Pisa University.

### Morphologic investigations

All the morphologic investigations following NAT or surgical excision (cytology and histology) were performed at the Institute of Pathology of the University Hospital of Pisa, where the specific diagnostic criteria have been described [5], [8]. Small (<1 cm) incidentally discovered carcinomas, metastatic nonthyroid carcinomas, and lymphomas were not considered in this study.

### Needle aspiration techniques (NAT)

#### FNAB

One mL or less of local anaesthetic (lidocaine 2%) was injected subcutaneously. All punctures were made through a single skin puncture moved over the nodule to the various sites to be aspirated using 23 or 22 gauge needles. Cytological smears were air dried for Giemsa staining or spray fixed for the Papanicolaou method.

#### LNAB

The LNAB technique was first reported in 1930 [9] and is very similar to that used for FNA. No incision of skin is performed; skin is anaesthetized as for FNAB. The syringe can contain heparin to prevent coagulation of blood around the tissue specimen. The needle is inserted into the nodule through the same skin puncture already made for the previous FNAB and is then rotated within the nodule so that the sharp end severs the tissue fragments, which are than aspirated into the barrel of the syringe. A simpler method to obtain tissue fragments is to perform the same procedure as for FNAB only more vigorously. Needles of different sizes can be used according to the dimensions and consistency of the nodule [10], [11]. Needle sizes range from 18 for the largest nodules (>35 mm) to 22 gauge for the smallest nodules (about 10 mm). LNAB provides tissue fragments of variable size depending on the needle size and the nodule pathology. The tissue fragments are easily visible [5], [12].

### Comparison studies

The first group included cancers with minimal preoperative selection and an extensive use of surgery, providing a reference TC proportional rate. This rate was compared with those observed in the other two groups when TN were selected with NAT.

### Statistical analysis

Data were expressed as percentages and the 95% confidence interval was computed by the Clopper-Pearson method. The comparison among groups was performed by the Fisher-Freeman-Halton's exact test. For statistical analysis StatXact-4.0.1 was used.

## Results

The clinical data of the 3 TN patient groups are given in Table 1. Patients were primarily from the central and southern regions of Italy.

**Table 1:** Incidence of thyroid cancer in patients with palpable thyroid nodules submitted to or selected for surgery*.

Years	Preoperative selection method	TN patients examined	TN patients operated	Cancers postoperatively detected	Cancers/examined patients**	Cancer histotype***				
		n	n	n	%	Pap n (%)	Foll n (%)	Med n (%)	Anapl n (%)	Other n (%)
1972–1979	Surgery	1140	1140	35	3.07	21 (60)	11 (31.4)	2 (5.7)	1 (2.8)	
1980–1992	NAT	5403	483	150	2.77	120 (80)	23 (15.3)	3 (2.0)	3 (2.0)	1 (0.7)
1993–2010	NAT	1568	143	46	2.93	41 (89.1)	4 (8.7)		1 (2.2)	

* NAT = needle aspiration techniques, TN = thyroid nodule, pap = papillary, foll = follicular, med = medullary, anapl = anaplastic.

** difference among groups: p = 0.810.

*** difference among groups: p = 0.008 for papillary histotype; p = 0.027 for follicular histotype.

From 1972 to 2010, 1,766 TN patients were operated on following two different selection criteria. A total number of 231 cancers were found at postoperative histology. This figure corresponds to 2.85% of all the preoperatively selected TN patients. The distribution of these thyroid cancers in the 3 periods considered was the following: 1972–1979, 35 cancers (papillary 21, follicular 11, medullary 2, and anaplastic 1) corresponding to 3.07% (95% CI: 2.15–4.24%) of all the preoperatively selected TN patients; 1980–1992, 150 cancers (papillary 120, follicular 23, medullary 3, anaplastic 3, and squamous 1) corresponding to 2.77% (95% CI: 2.35–3.25%) of all the preoperatively selected TN patients; 1993–2010, 46 cancers (papillary 41, follicular 4, anaplastic 1) corresponding to 2.93% (95% CI: 2.16–3.89%) of all the preoperatively selected TN patients. The percentage of TC was not significantly different in the 3 considered periods (Fisher-Freeman-Halton's exact test p = 0.810).

A significant change in the cancer histotype composition during the 3 considered time periods was observed. The papillary histotype significantly increased (p = 0.008) while the follicular histotype significantly decreased (p = 0.027).

## Discussion

This study shows that the proportional rate of TC among patients with a TN examined by the same physician within the University Hospital of Pisa and morphologically investigated at the same Institute of Pathology did not significantly change in the last 40 years. This consistency occurred even though the methods of cancer detection changed from minimal preoperative selection with extensive surgery to more aggressive selection by sensitive NAT. This observation is corroborated by the data from a different 34,266 TN patient series evaluated in a different Department of the University Hospital of Pisa from 1997 to 2004 [13]. In this study, there were 3,406 excised thyroid nodules with 1,208 thyroid cancers in 3,004 patients, of which 1062 had TC (3.1%). This proportional rate is very similar to that observed among our 3 study groups.

These two clinical series represent the principal source of data on the TC proportional rate among patients with TN within the University Hospital of Pisa. Our study first monitors the proportion of TC among TN in a consecutive long series of data from the same team. All the other studies are substantially cross-sectional and do not report any information on the time trend. During the study no significant change in the proportional rate of TC among TN patients occurred. This finding suggests that pathogenetic risk factors and more sensitive diagnostic technologies did not differentially affect the incidence of TC and TN.

In 2000, the TC incidence among European countries varied by a factor of 3–4 (from 2–3 to 8–9 per 100.000 women) with progressive increases in some countries (e.g., Finland and France) and no changes in others (Sweden and Norway) [1]. In the United States, Davies and Welch [2] reported that the incidence of TC more than doubled over the past 30 years (from 3.6 per 100.000 in 1973 to 8.7 per 100.000 in 2002) and that 87% of the increase was due to the diagnosis of small papillary cancers (≤1 cm). In Italy, Buzzoni [3] reported that from 1993–1995 to 2003–2005, the crude TC incidence rate increased 118% (from 6.5 to 14.2 cases for 100.000 inhabitants/years), and that the increase due to an aging population was only 6%. Similar TC incidence data in Italy were reported by Dal Maso et al. [14] when comparing the periods 1991–1995 and 2001–2005. Different TC incidence rates with similar selection protocols and technologies, or varying ages and study periods, raised the question of a changing carcinogenic environment [15].

Risk factors for TC [1] include exposure to ionizing radiation in childhood, family history, other thyroid diseases, dietary iodine, and environmental pollutants (endocrine disruptors). Only relatively high doses of ionizing radiations (>200 mSv) can significantly increase the TC incidence rate [1].

The presence of goiter, TN, hypothyroidism with elevated TSH, or autoimmune disease has been considered as an important risk for TC [16], [17]. Dietary iodine content is a factor affecting the occurrence of goiter or a TN and may therefore have indirect effects. Endocrine disruptors, such as iodine, polychlorobiphenyls, and some pesticides are suspected to be carcinogenetic for the human thyroid, though no conclusive evidence has been demonstrated [1].

Quantitative analysis has shown that, apart from childhood irradiation, a benign thyroid lesion is the strongest risk factor (RR 12–33) for TC [16], [17]. This suggests that cellular proliferation can be a mechanism common to many tumor processes as induced by radiation, TSH, low iodine [13]. It should be noted that any risk factor or process common to both benign and malignant TN may not influence the proportional changes between the two. This is a potential confounder to any conclusion that environmental factors have remained stable over the 40 year study period described herein.

Since the 1950 report of Duffly and Fitzgerald [18] there has been increasing awareness that radiation therapy to the upper body administered to infants, children, and even young adults may induce carcinoma of the thyroid 10 to 30 years later [1]. There was general agreement that the incidence of nodular thyroid disease was much higher in irradiated patients as compared with nonirradiated controls [18]. There was also general agreement that the percentage of malignant TN was similar in both irradiated and nonirradiated groups [19]. Thus, the aim of screening radiation-exposed individuals was to identify those individuals who were at high risk for developing nodular thyroid disease [19]. Our data are consistent with these conclusions that improved detection of any TN, as the strongest risk factor for TC, may be a more important contribution to the TC incidence than diagnostic procedures that identify high risk TN.

When the population exposed to atomic weapons testing in the Marshall Islands were examined approximately 35 years after, the proportional rate of TC among TN was 3.1% [20]. In this population, the proportion of TN patients detected with US was 21.2%. Our data are also consistent with this TC proportion [20].

Following the Chernobyl accident, highly irradiated (10.8 cGy) adults within the first 5 years showed a TN incidence of 1% using US [21]. Four years after the accident, Mettler et al. [22] reported that the prevalence and characteristics of TN in contaminated settlements and unexposed populations were the same (using US screening). In contrast, Drozd et al. [23] reported that among 1,132 highly irradiated children (4–14 years old when exposed and 8–18 when investigated), the proportion of TN patients and the proportion of TC among TN patients were 1.2% and 50%, respectively, much higher than in the control unexposed population where a few benign nodules and no cancer were observed. In 2 studies, each comparing relatively contaminated areas far from Chernobyl, with a control group in more distant uncontaminated areas, no difference was found in children or adolescents, with a ratio of TN subjects/investigated subjects varying from 2% to 6% [24], [25]. Among children and adolescents exposed (0.79 grays) to ^131^I after Chernobyl and evaluated 12 to 14 years later, TN was found in 2.7%, with 12% harboring TC [26]. Reiners reported a progressive increase of TC incidence in irradiated children and adolescents up to 10 or 15 years after the accident; however he did not describe TN incidence [27]. These studies suggest that age at the time of the investigations significantly affected TN incidence [22], [23]. However, although the general opinion is that ionizing radiation increases the number of both benign and malignant nodules [19], [20], [22], the Chernobyl data do not clarify whether at some time interval from the irradiation and at some young age, TC incidence rates increase more that those for benign nodules.

A noteworthy finding is that the proportion of follicular cancer was reduced from 31.4% to 8.7% during the study. Possible explanations for this finding are the following. At the beginning of the study some follicular variants of papillary cancers and some largely follicular differentiated cancers were diagnosed as follicular cancer while by the time these diagnoses changed into papillary cancer [5]. The iodine prophylaxis, which was more and more advised and promoted during the study, may have contributed to reducing follicular cancer [28], [29].

Molecular genetics has recently improved the understanding of TC pathogenesis [30] and may represent a useful investigative tool.
